# A global effort toward standards for data sharing in biomedical imaging: Developing Consensus and Infrastructure for Global Data Interoperability

**DOI:** 10.1038/s44319-025-00652-w

**Published:** 2025-12-03

**Authors:** Sophie L Winter, Josh Moore, Adriana A S Tavares, Graham Galloway, Michel Dojat, Dario Livio Longo, Ryan Sullivan, Aastha Mathur, Linda Chaabane

**Affiliations:** 1 Global BioImaging, EMBL Heidelberg, Heidelberg, Germany; 2 German BioImaging—Society for Microscopy and Image Analysis e.V., Constance, Germany; 3 Institute for Neuroscience and Cardiovascular Research, University of Edinburgh, Edinburgh, UK; 4 Edinburgh Imaging, University of Edinburgh, Edinburgh, UK; 5 Centre for Advanced Imaging, AIBN, The University of Queensland, Brisbane, QLD Australia; 6 Univ. Grenoble Alpes, Inserm U1216, Grenoble Institut Neurosciences, Inria, La Tronche, France; 7 National Research Council of Italy (CNR), Institute of Biostructures and Bioimaging (IBB), Turin, Italy; 8 National Imaging Facility, University of Sydney, Sydney, NSW Australia; 9 EURO_BIOIMAGING Bio-Hub, EMBL Heidelberg, Heidelberg, Germany; 10 EURO-BIOIMAGING Med-Hub - National Research Council of Italy (CNR), Institute of Biostructures and Bioimaging (IBB), Turin, Italy

**Keywords:** Methods & Resources, Science Policy & Publishing

## Abstract

This article examines the global challenges and emerging solutions in sharing and standardizing biomedical imaging data across modalities. It highlights international efforts to develop FAIR, interoperable frameworks that bridge biological and clinical imaging communities.

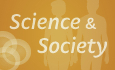

Since Wilhelm Röntgen discovered X-rays in 1895, imaging technologies have transformed the life sciences. These technologies—from early radiography and light microscopy to bioluminescent and fluorescent imaging to high-resolution microscopy—have enabled scientists to visualize cells, tissues and molecular processes in unprecedented detail. By making the microworld visible, imaging has accelerated breakthroughs across nearly every domain of science, from anatomy to physiology to genetics to disease research. Today, high-precision imaging modalities like MRI, CT, and advanced microscopy continue to drive innovation across biomedical research, pharmaceutical development, and biotechnology, generating vast and valuable datasets.

These images are collected from various subjects in different corners of the world, under varied conditions, with numerous instruments and protocols. Gathering and sharing this treasure trove of imaging data would unlock a wide range of transformative possibilities for science and medicine—for validating scientific findings, supporting research, accelerating AI-driven discovery, development of new diagnostic tools and models for disease stratifications and personalized treatments. If properly organized and shared, it could fuel reproducible research, accelerate discoveries, help uncover rare disease patterns, support regulatory decisions, and dramatically improve diagnosis and treatment. But this potential remains largely unrealized. Why?

“Gathering and sharing this treasure trove of imaging data would unlock a wide range of transformative possibilities for science and medicine…”

In an effort to understand the challenges of image-data management, particularly in the biomedical sciences, and identify obstacles to open access and globally shared standards, Global BioImaging’s biomedical expert group convened a panel discussion with experts from various imaging facilities around the world. Following the discussion with participants from 63 countries, the panelists and moderators who co-author this publication have identified key challenges and potential solutions outlined below.

## The challenge of building consensus

The current biomedical image data ecosystem is deeply fragmented. First, “imaging” comprises a vast number of technologies that are mostly coupled to different data-processing and analysis tools. Secondly, imaging data are often stored in siloed repositories, saved in a multitude of proprietary formats and annotated using inconsistent terminology—or not annotated at all. Third, researchers must handle massive volumes of data across multiple biological scales, from molecules to whole organisms, while ensuring interoperability with other data types such as genomics or clinical records. Additionally, privacy regulations, inconsistent standards and the need for high-quality annotations complicate data sharing and AI-driven analysis. Especially in medical imaging, patient privacy and data anonymization are critical and global sharing of imaging data is hindered by regulatory and ethical constraints. The lack of standardization across institutions creates further barriers to access and reuse.

Even when valuable datasets exist, it is often unclear how to interpret or build upon them, because their metadata representation—the structured description of what the image shows, how it was created, processed and analyzed—is often incomplete or inconsistent. As Josh Moore, technical director at German BioImaging, put it during the panel discussion: “Creating metadata representation is easy. The hard part is building consensus,” which has been his and others’ goal with the Open Microscopy Environment (OME) since its inception in 2003 (Swedlow et al, 2003).

“Even when valuable datasets exist, it is often unclear how to interpret or build upon them, because their metadata representation […] is often incomplete or inconsistent.”

As the critical missing piece, consensus is perhaps also the hardest to achieve. As Moore explained, aligning on common terms, standards, and formats is essential if we want imaging data to be FAIR (Findable, Accessible, Interoperable, Reusable) (Swedlow et al, 2021). But these discussions often stall due to a lack of structured leadership and well-defined goals. “You need a definition of ‘done’,” Moore said, “because these conversations can go in circles. And they burn people out”.

The challenge becomes even more pronounced in preclinical imaging, where infrastructure and metadata standards are underdeveloped. As Dario Longo, senior researcher at the National Research Council of Italy, pointed out, “We don’t have a common metadata model to describe a preclinical image dataset. Even when initiatives exist, they’re often limited to single institutions or regional efforts. That severely restricts data sharing and reuse.”

So, how to move forward? The path begins with shared ontologies—common terminologies that describe datasets in consistent, machine-readable ways. It continues with the design of tools and platforms that make sharing easy. Ultimately, it requires a cultural shift: toward open science, collaborative infrastructure and a collective understanding that data are more valuable when shared.

## Current landscape and progress

During the past decade, several national, European and global initiatives—such as Euro-BioImaging, Global BioImaging, the Australian Imaging Service (AIS), and the European Open Science Cloud (EOSC)—have been building the digital and human infrastructure to make imaging data FAIR. These efforts were also driven by large funding agencies that increasingly require data-management plans and open-science principles.

Despite this progress, biomedical imaging still lags behind disciplines such as genomics or proteomics, where global standards and centralized repositories are well established. There is currently no universally adopted framework for data sharing, and many imaging communities continue to rely on local or ad hoc systems. This gap is largely a consequence of the rapid technological evolution in imaging—data volume, modality diversity and analytical complexity have all expanded far faster than the organizational structures to manage them.

“There is currently no universally adopted framework for data sharing, and many imaging communities continue to rely on local or ad hoc systems.”

Complementing large-scale infrastructure initiatives, the BioImage Archive (BIA; Hartley et al, 2022), hosted by EMBL-EBI, provides a central repository for biological imaging data across multiple modalities—from light and electron microscopy to X-ray tomography. By enabling researchers to upload, share and reuse published datasets, BIA exemplifies how FAIR data principles can be implemented in practice. It integrates with related resources such as the Electron Microscopy Public Image Archive (EMPIAR; Iudin et al, 2023) and the Image Data Resource (IDR; Williams et al, 2017), to promote interoperability and consistent metadata annotation through REMBI standards.

However, BIA’s scope is explicitly limited to non-clinical imaging and excludes patient-identifiable data derived from medical imaging modalities such as PET/SPECT, CT or MRI. This focus reflects both privacy considerations and the mission to support research at molecular, cellular, and organismal scales rather than diagnostic medicine.

Another important factor is the open microscopy environment (OME; Linkert et al, 2010), an open-source software and data-model initiative that defines standardized file formats—most notably OME-TIFF—and a unified metadata model for image data. OME’s tools, including OMERO and Bio-Formats, enable researchers to store, visualize and analyze data across instruments and formats, fostering interoperability within microscopy and other imaging domains. While Bio-Formats technically supports a wide range of file types, including some used in medical imaging, OME is mostly adopted in light and fluorescence microscopy workflows. Clinical biomedical imaging fields such as radiology and nuclear medicine typically rely on different standards, particularly DICOM, which is deeply embedded in hospital and regulatory infrastructures.

This distinction underscores a key challenge: successful frameworks for biological imaging do not yet have direct equivalents in biomedical imaging. The latter faces additional barriers, including data privacy regulations, ethical governance and the need for large-scale harmonization across hospital, research and industrial contexts.

Several targeted initiatives, particularly in the medical domain, are beginning to address aspects of this fragmentation but remain focused on specific applications. The Cancer Imaging Archive (TCIA) in the USA has become a widely used repository for clinical imaging datasets, especially in oncology. The European Health Data Space (EHDS) aims to enable secure cross-border exchange of health data, including imaging, with a strong emphasis on patient privacy and ethical use. In that direction, the infrastructure, called Cancer Image Europe, is currently designed to provide a secure platform to facilitate access to diverse clinical image datasets and tools to accelerate the development of innovative solutions for cancer diagnosis and treatment (https://cancerimage.eu/). On the technical side, QUAREP-LiMi—while centered on light microscopy—demonstrates how community-driven efforts can successfully establish quality control and metadata standards that may serve as models or building blocks for other imaging domains.

“Several targeted initiatives, particularly in the medical domain, are beginning to address aspects of this fragmentation but remain focused on specific applications.”

These efforts, while promising, remain largely disconnected, though. Bridging them will require not only technical solutions but also cultural and institutional alignment, beginning with consensus on how to describe, store and share imaging data in ways that are meaningful across disciplines.

## Key challenges in a nutshell

These limitations converge into several major challenges that must be addressed in order to build a coherent, scalable imaging-data infrastructure.

Interoperability gaps: There is a lack of standardized metadata and ontologies across platforms, making it difficult to search, compare or aggregate datasets. Pipelines and search tools that can operate across repositories are still limited in scope and adoption. Despite the rapid development of AI tools to navigate complex datasets without universal standards, human review is critical and relies on standardized data annotations.

Data-sharing bottlenecks: Cultural barriers remain a powerful deterrent. Many researchers question the value of sharing their data, especially when career incentives favor publications over building infrastructure. Tools for uploading, annotating and accessing data are often too technical or cumbersome. Data stewardship is usually underdeveloped, with individual researchers left to create data plans from scratch without institutional guidance.

User experience difficulties: Researchers face a steep learning curve when navigating heterogeneous repositories. Poorly annotated datasets and non-standard formats lead to inefficiencies or outright data loss. Incentives to properly manage and maintain datasets are often lacking, resulting in the long-term deterioration or disappearance of valuable imaging data. Specific training on data management is not commonly provided in most research institutions. In biomedical imaging, this is further complicated by the requirements of ethics, clinical governance and privacy. For many researchers, it is easier to store data in a local, private storage, rather than navigating the ethical requirements of sharing it.

“For many researchers, it is easier to store data in a local, private storage, rather than navigating the ethical requirements of sharing it.”

Financial sustainability: Sustainable funding and long-term maintenance remain uncertain for many platforms. Without stable funding mechanisms, many promising repositories struggle to stay online, let alone scale globally.

## Emerging solutions: national perspectives

Despite these challenges, national and international initiatives are beginning to chart a path toward more cohesive imaging-data sharing. To illustrate how these challenges are being tackled in practice, the workshop panelists presented national perspectives from several key initiatives.

The Australian Imaging Service (AIS) is a national—and increasingly international—platform to enhance research reproducibility and accessibility of trusted image analysis techniques. Funded by the Australian Government, AIS brings together browser-based secure data-management and analysis tools that allow researchers, even without informatics expertise, to process and analyze imaging data reproducibly. Established in 2021, AIS now comprises 13 institutional nodes supporting more than 460 users across Australia, the EU and the USA. Each node integrates long-term data storage and computing resources connected to imaging instruments within universities and hospitals, enabling analysis where the data reside.

Using a data-centric computing model built on XNAT (Extensible Neuroimaging Archive Toolkit, an open-source platform for storing, managing and processing biomedical imaging data) and Kubernetes (an open-source system for orchestrating containerized applications, automation deployment, scaling and management), AIS provides automated pipelines for data conversion and quality control, virtual desktops for real-time visualization and federated machine-learning tools that support privacy-preserving analysis. The platform’s curated library of datasets and containerized tools ensures full software reproducibility across sites. Current exemplar projects include an international multiple sclerosis imaging repository and a national melanoma program linking 16 hospitals. By harmonizing infrastructure and standards across institutions, AIS demonstrates how coordinated, secure and sustainable national systems can support FAIR imaging data practices and foster global collaboration.

France has also made significant strides in addressing the technical and organizational challenges of biomedical image-data management. A prime example is the FLI-IAM platform, developed as part of the France Life Imaging (FLI) initiative—a national effort to boost data sharing in clinical and preclinical studies across research centers (Commowick et al, 2021; Kain et al, 2020; Commowick et al, 2018).

As Michel Dojat, Neuroimaging Expert and coordinator of FLI’s imaging-data infrastructure, explained, the goal of FLI-IAM is to make both clinical and preclinical imaging data more accessible and reusable by providing an integrated, secure system for storage, management and processing. Through the FLI-IAM web portal, researchers can explore available studies, apply for data access, and, once approved, download imaging datasets (https://shanoir.irisa.fr/shanoir-ng/welcome). The platform brings together two key tools to ensure seamless communication and maintain the link between raw and processed data. Shanoir provides secure management of imaging datasets at the study level—including upload, download, quality control and visualization—all underpinned by a dedicated ontology to promote interoperability across datasets (Batrancourt et al, 2015; Temal et al, 2008). The other tool is VIP (Virtual Imaging Platform), an online portal that enables researchers to run standardized image processing pipelines, such as FSL or SPM12, for brain imaging studies, as well as custom workflows encapsulated using Docker technology.

FLI-IAM also promotes interoperability beyond national borders. “We want to make our platform interoperable with other repositories in Europe,” Dojat emphasized, highlighting collaborations with, for example, the open microscopy environment OMERO and genetic databases. This integration allows researchers to associate imaging data with processing tools and even assign digital object identifiers (DOIs), ensuring that datasets and tools can be properly cited and reused in future studies. This integrated, scalable model demonstrates how national infrastructure can foster reproducibility, interoperability and responsible research—not only within France but as part of the broader international push toward open, FAIR imaging data.

The Scottish Data Haven, operated through Research Data Scotland, is designed as an end-to-end platform providing researchers with access to routinely collected health data, including medical imaging. At its core lies the Scottish Medical Imaging Archive (Baxter et al, 2024), which contains more than 57.3 million radiology studies, each connected to corresponding medical records—a resource of unprecedented scale.

As Adriana Tavares, Head of the Preclinical PET Facility at the University of Edinburgh and Professor of Translational Molecular Imaging, explained during a recent panel discussion: “The whole project took over 7 years—designing the platform, building the software, integrating all these data resources—a collection of population-based and routinely collected medical radiology images”. The scale and ambition of the project reflect both the complexity and the potential of national-level solutions.

Tavares emphasized that data management in this context is not only a scientific requirement—it is also an ethical responsibility (Bauchner et al, 2016). Historical evidence shows that existing data sharing practices often fall short (Tederso et al, 2021), a gap that has motivated funders to invest in infrastructure that enables the ethical and efficient reuse of already acquired imaging data, for example, through initiatives such as the Wellcome Trust longitudinal population studies award.

The Scottish Data Haven follows a secure enclave model, one of several established approaches to enable data sharing. These models generally fall into three categories: distributed data analysis, where only anonymous, aggregated data are exchanged; secure multi-party computation, which allows encrypted data to be analyzed without direct access to raw data; and data enclaves, which store pooled, individual-level data in secure environments accessible to approved researchers (Wirth et al, 2021).

The platform is now approaching full self-sustainability, having transitioned from its origins as a multi-million-pound publicly funded initiative over the past 7–8 years to a fee-for-service model that supports ongoing operations. “The idea is that moving forward, this will be provided as a service—an end-to-end, open-source software platform to host and manage data— making secure data access a reality for researchers,” Tavares explained.

The EU-funded project foundingGIDE (https://founding-gide.eurobioimaging.eu/)is a community-driven effort aimed at tackling one of the biggest challenges in the life sciences today: making vast and complex imaging data interoperable. It builds consensus by coordinating community efforts around standardizing biological and preclinical metadata and ontologies, starting from a high level and translating this into practical solutions. One of the project’s main aims is to facilitate metadata harmonization across representative image repositories. For bioimaging data, this includes the three major biological image data repositories BIA, IDR and SSBD to enhance data discoverability, searchability and reuse.

For the preclinical imaging community, the project aims to provide recommendations on ontologies, a definition of a metadata model for describing preclinical image datasets and providing a platform (PIDAR—Preclinical Image DAtaset Repository, https://pidar.hpc4ai.unito.it/) for improving findability and reusability. XNAT, an open-source platform for storing, managing and processing biomedical imaging data, initially developed at The University of Washington (Marcus et al, 2007), forms the backbone of foundingGIDE’s plans for biomedical image data storage. It has become an open-source imaging informatics platform and is used widely in the biomedical research community, including the Australian Imaging Service. Often thought to be specific for DICOM, it has grown beyond that, storing all major image formats (NIFTI, BIDS, MINC) as well as MR Spectroscopy, histology, dermatology and non-image clinical and genomic data. In addition, tools as XNAT-PIC for facilitating the use of the XNAT platform for uploading, labeling and processing biomedical image datasets have been developed by Euro-BioImaging Medical Hub for the preclinical researchers’ community (Zullino et al, 2022).

Central to foundingGIDE’s approach is “active community engagement through hands-on workshops, community events and hackathons” according to its Scientific Coordinator, Aastha Mathur, Head of Image Data Services at Euro-BioImaging (https://www.eurobioimaging.eu/image-data-services/). The initiative brings together global imaging experts, policymakers, funders, and imaging and data researchers to tackle key challenges, and explore innovative pathways toward a truly interoperable global image data ecosystem (GIDE). These activities help to ensure that recommended solutions reflect the needs of the diverse imaging community. By focusing on practical interoperability, foundingGIDE stands as a proof of concept of how inclusive collaboration can create impactful results.

## Conclusion

Biomedical research increasingly depends on our ability to share, integrate and analyze imaging data at scale. Achieving this vision will require more than infrastructure—it will demand consensus, collaboration and a renewed commitment to open-science values.

Moving toward global standards and shared ontologies will require a coordinated effort among international imaging infrastructures, standards initiatives and domain-specific scientific societies. These groups could act jointly as a global imaging standards forum, supported by funders and policy agencies to define, test and maintain reference ontologies and metadata models across modalities. Establishing such a framework—community-driven yet formally recognized—could ensure that imaging data standards evolve in step with technology, and are adopted widely and sustainably.

“Moving toward global standards and shared ontologies will require a coordinated effort among international imaging infrastructures, standards initiatives, and domain-specific scientific societies.”

## Supplementary information

Peer Review File

## References

[CR1] Batrancourt B, Dojat M, Gibaud B, Kassel G (2015) A multilayer ontology of instruments for neurological, behavioral and cognitive assessments. NeuroInformatics 13:93–110 25240319 10.1007/s12021-014-9244-3 PMC4303739

[CR2] Bauchner H, Golub RM, Fontanarosa PB (2016) Data sharing. an ethical and scientific imperative. JAMA 315:1238–1240 10.1001/jama.2016.2420 27002444

[CR3] Baxter R et al (2024) The Scottish medical imaging archive: 57.3 million radiology studies linked to their medical records. Radiol Artif Intell 6:e220266 38166330 10.1148/ryai.220266 PMC10831519

[CR4] Commowick O et al (2018) Objective evaluation of multiple sclerosis lesion segmentation using a data management and processing infrastructure. Sci Rep 8:13650 30209345 10.1038/s41598-018-31911-7 PMC6135867

[CR5] Commowick O et al (2021) Multiple sclerosis lesions segmentation from multiple experts: the MICCAI 2016 challenge dataset. Neuroimage 244: 118589 34563682 10.1016/j.neuroimage.2021.118589

[CR6] Hartley M, Kleywegt GJ, Patwardhan A, Sarkans U, Swedlow JR, Brazma A (2022) The bioimage archive – building a home for life-sciences microscopy data. J Mol Biol 434: 167505 35189131 10.1016/j.jmb.2022.167505

[CR7] Iudin A, Korir PK, Somasundharam S, Weyand S, Cattavitello C, Fonseca N, Salih O, Kleywegt GJ, Patwardhan A (2023) EMPIAR: the electron microscopy public image archive. Nucl Acids Res 51:D1503–D1511 36440762 10.1093/nar/gkac1062 PMC9825465

[CR8] Kain M et al (2020) Small animal shanoir (SAS): a cloud-based solution for managing preclinical MR brain imaging studies. Front Neuroinformatics 14:20 10.3389/fninf.2020.00020 PMC7248267 32508612

[CR9] Linkert M et al (2010) Metadata matters: access to image data in the real world. J Cell Biol 189:777–782 20513764 10.1083/jcb.201004104 PMC2878938

[CR10] Marcus DS, Olsen T, Ramaratnam M, Buckner RL (2007) The extensible neuroimaging archive toolkit (XNAT): an informatics platform for managing, exploring, and sharing neuroimaging data. Neuroinformatics 5:11–34 17426351 10.1385/ni:5:1:11

[CR11] Swedlow JR, Goldberg I, Brauner E, Sorger PK (2003) Informatics and quantitative analysis in biological imaging. Science 300:100–102 12677061 10.1126/science.1082602 PMC3522889

[CR12] Swedlow JR, Kankaanpää P, Sarkans U, Goscinski W, Galloway G, Malacrida L, Sullivan RP, Härtel S, Brown CM, Wood C, Keppler A, Paina F, Loos B, Zullino S, Longo DL, Aime S, Onami S (2021) A global view of standards for open image data formats and repositories. Nat Methods 18:1440–1446 33948027 10.1038/s41592-021-01113-7

[CR13] Tederso L et al (2021) Data sharing practices and data availability upon request differ across scientific disciplines. Sci Data 8:192 34315906 10.1038/s41597-021-00981-0 PMC8381906

[CR14] Temal L, Dojat M, Kassel G, Gibaud B (2008) Towards an ontology for sharing medical images and regions of interest in neuroimaging. J Biomed Inf 41:766–778 10.1016/j.jbi.2008.03.002 18440282

[CR15] Williams E et al (2017) Image data resource: a bioimage data integration and publication platform. Nat Methods 14:775–781 28775673 10.1038/nmeth.4326 PMC5536224

[CR16] Wirth FN, Meurers T, Johns M, Prasser F (2021) Privacy-preserving data sharing infrastructures for medical research: systematization and comparison. BMC Med Inform Decis Mak 21: 242 34384406 10.1186/s12911-021-01602-x PMC8359765

[CR17] Zullino S, Paglialonga A, Dastrù W, Longo DL, Aime S (2022) XNAT-PIC: extending XNAT to preclinical imaging centers. J Digit Imaging 35:860–875 35304674 10.1007/s10278-022-00612-z PMC9485318

