# Supplementary material for: A global effort toward standards for data sharing in biomedical imaging: Developing Consensus and Infrastructure for Global Data Interoperability
Source: EMBO Rep. 2025 Dec 3;27(1):10–4. doi: 10.1038/s44319-025-00652-w (PMC12796427; doi:10.1038/s44319-025-00652-w)
Supplement: Supplementary file 1 — Peer Review File [file 44319_2025_652_MOESM1_ESM.pdf]

EMBO reports

A Global Effort Toward Standards for Data Sharing in Biomedical Imaging

Sophie Winter, Josh Moore, Adriana Tavares, Graham Galloway, Michel Dojat, Dario Livio Longo, Ryan Sullivan, Aastha Mathur, and Linda Chaabane

Corresponding authors: Linda Chaabane (linda.chaabane@eurobioimaging.eu) , Sophie Winter (sophie.winter@embl.de)

Review Timeline:

Submission Date:	7th Nov 25
Accepted:	11th Nov 25

Editor: Holger Breithaupt

Transaction Report:

This manuscript was editorially reviewed.
